# Genetic analysis of the forkhead transcriptional factor 2 gene in three Chinese families with blepharophimosis syndrome

**Published:** 2013-02-20

**Authors:** Haiou Jiang, Xueshuang Huang, Zhiguang Su, Libing Rao, Sisi Wu, Ting Zhang, Kang Li, Qingli Quan, Kang Zhang

**Affiliations:** 1 Molecular Medicine Research Center, West China Hospital, Sichuan University, Chengdu, P. R. China; 2 Department of Medical Genetics, Huaihua Medical College, Hunan Province, Huaihua, P. R. China; 3 Institute for Genomic Medicine, and Shiley Eye Center, University of California, La Jolla, CA

## Abstract

**Purpose:**

Clinically, blepharophimosis syndrome (BPES) has been divided into two subsets according to the association of ocular malformation with (type I) or without (type II) premature ovarian failure (POF). BPES is ascribed to mutations in the forkhead transcriptional factor 2 (*FOXL2*) gene. This study aimed at identifying clinical features and mutations within the *FOXL2* gene in three Chinese families with BPES.

**Methods:**

A clinical and molecular genetic investigation was performed in affected and unaffected members from three Chinese families with BPES. Genomic DNA was prepared from leucocytes of peripheral venous blood, the entire coding region of *FOXL2* were amplified with PCR, and direct DNA sequencing of the PCR products was performed for mutations in *FOXL2*.

**Results:**

Three mutations in *FOXL2* were found in three families, including c.672_701dup30, c.663_692dup30, and c.475dupC. Of the three, the c.475dupC (p.His159fs) was novel in family C and resulted in a frameshift mutation to generate a truncated protein owing to a premature stop codon at codon 238. The new duplication mutation was associated with BPES type II. The c.672_701dup30 (p.Ala224_Ala234dup10) and the c.663_692dup30 (p.Ala221_Ala231dup10) were detected in family A and family B, respectively, leading to expansions of the polyalanine (poly-Ala) tract that is frequently the hot spot of mutations within *FOXL2*.

**Conclusions:**

Our results expand the spectrum of *FOXL2* mutations, and further indicate the association of a novel duplication mutation leading to a truncated protein with BPES type II. The other two known mutations may support the previous hypothesis regarding expansions of the polyalanine tract associated with BPES type II as a mutational hot spot in *FOXL2*.

## Introduction

Blepharophimosis syndrome (BPES) is a rare autosomal dominant disorder with a prevalence of about 1 in 50,000 characterized by short palpebral fissures, epicanthus inversus, ptosis of the eyelids, and additional ocular and non-ocular features [1]. Two clinical types of BPES have been distinguished. In type I, eyelid abnormalities are associated with premature ovarian failure (POF), resulting in affected women without fertility, but in type II only the eyelid defects are found [2]. Both types of BPES are caused by mutation of the *FOXL2* gene [3,4], located at 3q23, which encodes a forkhead transcription factor containing a 100 amino acid DNA-binding forkhead domain and a polyalanine domain of 14 alanines.

Thus far, 106 unique intragenic *FOXL2* mutations have been identified in 206 unrelated families with BPES from different ethnic origins [5,6]. The previous studies demonstrated the existence of two mutational hotspots and showed genotype–phenotype correlations for a subset of intragenic mutations. Mutations predicted to result in a truncated protein before the polyalanine (poly-Ala) tract of the *FOXL2* gene may lead to BPES type I, whereas expansions of the poly-Ala tract from 14 to 24 alanine residues represent about 30% of all the mutations reported in the open reading frame of the gene and lead mainly to BPES type II [4,6,7]. However, the genotype–phenotype correlations for missense mutations and mutations leading to a truncated or extended protein containing an intact forkhead domain and poly-Ala tract remain elusive as they have been found in both types of BPES and manifest intra- and interfamilial variability [7,8].

In this study, we report three mutations identified in the *FOXL2* gene from three Chinese families with BPES. A novel duplication mutation leading to a truncated protein was associated with BPES type II.

## Methods

### Patients

**Figure 1:**
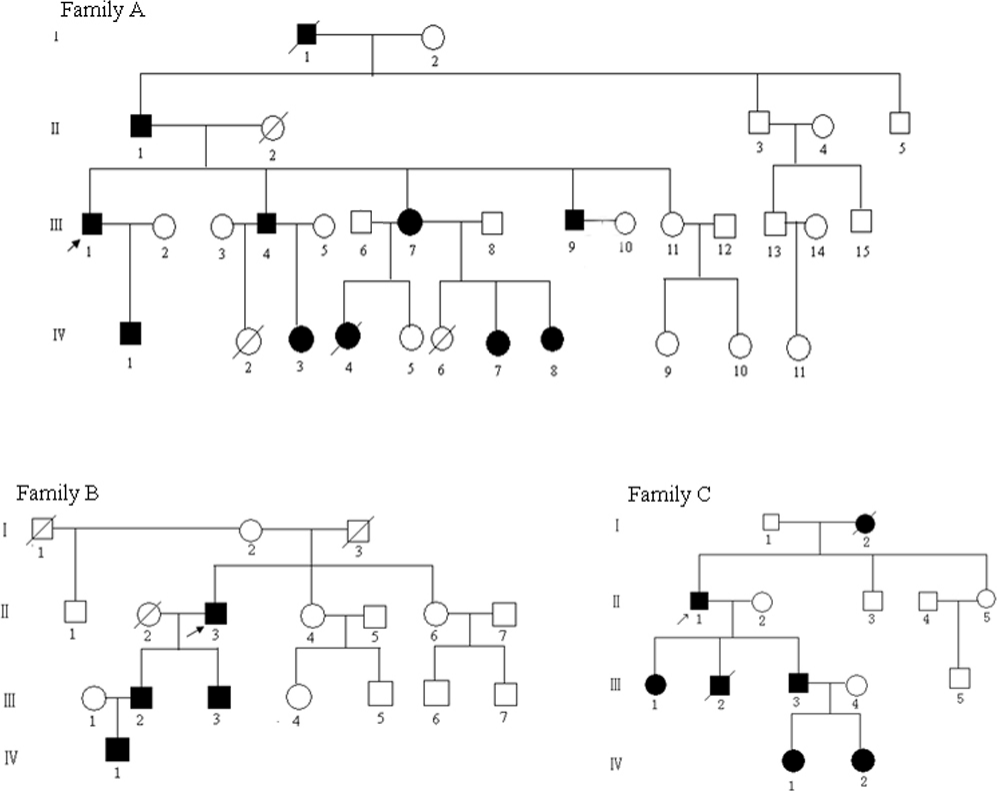
Pedigrees of three Chinese families. The closed symbols represent subjects with blepharophimosis syndrome (BPES), and the open symbols represent those who were unaffected. Arrows show the probands.

Eighteen affected (seven females and eleven males) and 37 unaffected individuals, with ages ranging from 2 to 73 years old, from three unrelated Chinese families in Hunan Province in China were enrolled in this study. In addition to the typical ocular manifestations of BPES, all patients in the three families had no other systemic diseases at the time of recruitment. No consanguineous marriage was observed in these families (Figure 1). The study was approved by Ethics Committee of West China Hospital, Sichuan University, Chengdu, China. Informed consent and permission to use patient photos in this report were obtained from all participants according to the principles of the Declaration of Helsinki.

### Clinical examination

Clinical examinations of all patients were performed by an experienced ophthalmologist. The BPES patients were diagnosed according to the criteria previously established with exclusion of microophthalmia. In brief, the patient’s facial picture presents four diagnostic criteria of BPES, including blepharophimosis, ptosis, epicanthus inversus, and telecanthus; and the patient meets at least three of the four diagnostic criteria of BPES mentioned on the clinical questionnaire. POF was defined as amenorrhea for a duration of ≥6 months at the age <40 years and a concentration of follicle-stimulating hormone (FSH) of > 40 IU/l.

### Molecular genetic analysis

Genomic DNA was prepared from leucocytes of peripheral venous blood of the patients and all relatives of the affected families as well as 100 randomly selected healthy volunteers, using a genomic extraction kit (Tiangen Biotech Co. Ltd, Beijing, China) according to the manufacturer’s instructions. DNA integrity was evaluated with 1% agarose gel electrophoresis. Amplification of the genomic fragments encompassing *FOXL2* coding regions (NCBI human genome build 35.1, NC_000003 for gDNA, NM_023067 for mRNA, and NP_075555 for protein) was conducted with PCR using the following primers: AF: 5′-GAA CTC GGT GGA GCC CAT ACG-3′, AR: 5′-GTA GAT GCC GGA CAG CGT GAG-3′, BF: 5′-CGG AGA AGA GGC TCA CGC TGT-3′, BR: 5′-CTC CCA GGC CAT TGT ACG AGT T-3′, CF: 5′-CCT GCA GTC TGG CTT CCT CAA-3′, CR: 5′-GGG ACA AAG AGG AGC GAC AGG-3′. The sizes of the PCR products were 683 bp, 624 bp, and 736 bp, respectively.

PCR was carried out in 30 μl reaction mixtures containing 40 ng genomic DNA, 1.0 μM of each of the forward and reverse primers, and 15 μl of 2X Taq Master Mix (SinoBio Biltech Co. Ltd, Shanghai, China). Thermocycling was conducted using the following program: initial denaturation at 95 °C for 2 min followed by 35 cycles of 94 °C for 10 s, 61 °C to 63 °C for 30s, and 72 °C for 1 min, and final extension at 72 °C for 5 min.

PCR products were purified with a cycle-pure kit (OMEGA; Bio-Tek, Doraville, GA) and sequenced using an ABI377XL automated DNA sequencer (Applied Biosystems, Foster City, CA). Sequence data and the published *FOXL2* consensus sequences were inputted in the SeqMan II program of the Lasergene package (DNAStar Inc., Madison, WI) and then aligned to identify variations. Each mutation was further validated with bidirectional sequencing in all relatives of the affected families and 100 randomly selected healthy volunteers. Mutation was described based on the nomenclature recommended by the Human Genomic Variation Society (HGVS).

## Results

### Clinical findings

**Figure 2:**
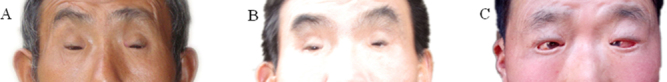
Eyelid photographs from three Chinese families with blepharophimosis syndrome (BPES): a bilateral reduction in horizontal fissure length, short palpebral width, and epicanthus inversus are seen in the patients. **A**, **B**, and **C** demonstrate the eyelid feature of the probands in family A, family B, and family C, respectively.

All patients in the three families were found to have typical features of BPES, including small palpebral fissures, ptosis of the eyelids, and epicanthus inversus (Figure 2). The segregation of the ocular anomaly in the three families was consistent with an autosomal dominant inheritance. The ocular anomaly of family A and family C was diagnosed as BPES type II due to female patients without POF, whereas the clinical subtype of family B remains unknown because there was no female patient in this pedigree. Detailed clinical data for each family’s proband are shown in Table 1.

**Table 1:** Clinical features of three probands from Chinese families with BPES

BPES families	Subtype	Affected individuals	Age (years)	IICD (mm)	IPFH (mm)		HPFL (mm)		Levator function (mm)		Surgical stages (preoperatively)
					RE	LE	RE	LE	RE	LE	
family A	II	III:1	47	38	2	3	22	22	2	2	Stage 1
family B	unknown	II:3	50	39	3	3	21	21	2	2	Stage 1
family C	II	II:1	63	36	4	4	23	23	2	2	Stage 2

Abbreviations: IICD, inner intercanthal distance; IPFH, vertical interpalpebral fissure height; HPFL, horizontal palpebral fissure length; LE, left eye; RE, right eye.

### Forkhead transcriptional factor 2 mutation identification and analysis

**Figure 3:**
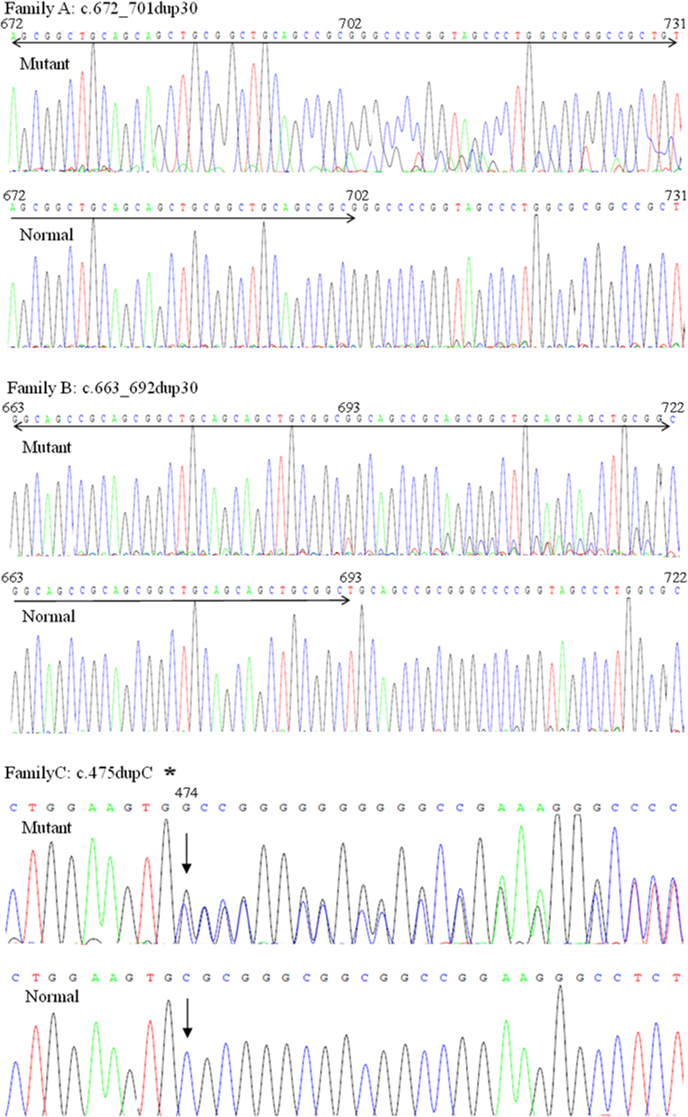
Sequence chromatograms of the mutations. The mutant alleles identified in the present study were compared with the normal alleles. The asterisk shows the reverse DNA sequence chromatograms.

Upon complete sequencing analysis of *FOXL2*, three heterozygous mutations were identified in three probands of the three families with BPES, including c.672_701dup30 (p.Ala224_Ala234dup10), c.663_692dup30 (p.Ala221_Ala231dup10), and c.475dupC (p.His159fs; Figure 3). Of the three, c.475dupC found in family C was novel. These mutations were also present in affected patients from corresponding families, but none of the unaffected family members and 100 normal control subjects examined carried these mutations.

The previously reported c.672_701dup30 (p.Ala224_Ala234dup10) mutation and c.663_692dup30 (p.Ala221_Ala231dup10) mutation were frameshift mutations inside the coding region for the polyalanine tract, leading to the poly-Ala expansions. This novel c.475dupC (p.His159fs) mutation between the DNA-binding forkhead domain and the polyalanine domain was also a frameshift mutation expected to cause miscoding of 79 amino acids from codon 159 and eventually a premature stop codon at 238. This change would generate a truncated protein.

## Discussion

The human *FOXL2* gene (OMIM 605597) codes a protein with 376 residues, which contains a DNA-binding forkhead domain (residues 52–152) and a polyalanine domain (residues 221–234) [3,9,10]. De Baere et al. classified the *FOXL2* mutations into seven groups (A–G) according to their effect on the predicted protein. Groups A–D contain the predicted truncated proteins: without a forkhead domain (group A), with a partial forkhead domain (group B), with a complete forkhead domain and without a poly-Ala tract (group C), and with complete forkhead and poly-Ala domains (group D). Group E comprises frameshift mutations that lead to elongated proteins with complete forkhead and poly-Ala domains. Group F contains in-frame changes, and group G contains missense mutations [7]. Of the three mutations identified in this study, a novel c.475dupC mutation between the DNA-binding forkhead domain and the polyalanine domain resulting in a truncated protein with a complete forkhead domain and without a poly-Ala tract belongs to group C, while the previously reported c.672_701dup30 mutation and c.663_692dup30 mutation inside the coding region for the polyalanine tract belong to group F, leading to the poly-Ala expansions. The exact function of polyalanine tracts in proteins is unknown, but the mechanism for the molecular pathogenesis of the poly-Ala expansions in BPES has been suggested to be a result of cytoplasmic aggregation of the *FOXL2* protein and inclusion in nuclear aggregates [4,11,12].

**Figure 4:**
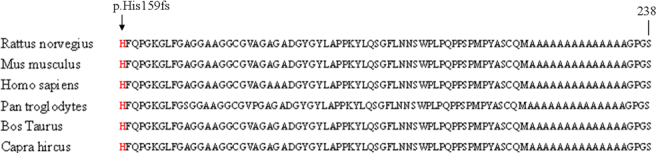
Multiple sequence alignment of the forkhead transcriptional factor 2 (FOXL2) protein region from residue His159 to Ser238. ClustalW analysis demonstrates that all amino acids in these successive positions are well conserved in orthologs. The new duplication mutation between the forkhead domain and the polyalanine domain is indicated with an arrow.

The novel c.475dupC (p.His159fs) mutation in family C was identified first in a Chinese family with BPES. The mutation had a C duplication at position 475, resulting in a frameshift producing 79 novel amino acids and terminating prematurely at codon 238. The mutation led to a truncated protein in which the entire polyalanine domain was erased. It has been shown that *FOXL2* mutations resulting in truncated proteins are associated with type I BPES while those causing extended proteins lead to type II BPES [3,4]. However, this genotype–phenotype correlation might not be general because of existing intra- and interfamily phenotypic variations [3,4,7,13,14]. In our study, the patients from family C with novel c.475dupC mutation, leading to a truncated protein, had type II BPES due to the affected women with fertility. Though we do not know exactly the mechanism by which the novel duplication mutation leading to a truncated protein is associated with BPES type II at present, by analyzing orthologs from six different species using the ClustalW tool online (Figure 4), we discovered all amino acids in the successive positions from codon 159 to 238 are highly conserved for *FOXL2*. This may reveal a potentiality that the region containing the c.475dupC mutation is important for *FOXL2* function. In addition, we found a c.663_692dup30 (p.Ala221_Ala231dup10) in the *FOXL2* gene, a hotspot mutation that caused poly-Ala expansions in family B, who include four generations with BPES. Interestingly, the four patients in this family were all male, which made it difficult to reach an early diagnosis of the BPES type because this variant was not present in the female members of this family. This mutation has been reported as a pathogenic change of type II BPES seven times (Human FOXL2 Mutation Database). Therefore, we predict that if a female patient occurs in this family, there will not be a risk of POF. Previous studies suggested that the c.672_701dup (p.Ala224_Ala234dup) hotspot mutation causing polyalanine expansion most likely leads to BPES type II [14]. In this study, the c.672_701dup identified in family A with type II BPES was in agreement with previous mutation studies of BPES.

In conclusion, we found a novel and two known mutations in the *FOXL2* gene from three large Chinese families with BPES. In this study, we report the association of a novel duplication mutation leading to a truncated protein with BPES type II. Our findings have widened the spectrum of *FOXL2* mutations in BPES and validated the mutation hotspot in *FOXL2*.
